# Editorial: Integrative microbial and chemical genomics to decipher antibiotic resistance mechanisms and developing innovative antimicrobial approaches

**DOI:** 10.3389/fmicb.2026.1938175

**Published:** 2026-08-04

**Authors:** William Calero-Cáceres, Bimal Jana, João Pedro Rueda Furlan, Juan C. Ortiz-Marquez

**Affiliations:** 1 Biotechnology Program, Department of Food and Biotechnology Science and Engineering, Universidad Técnica de Ambato, Ambato, Ecuador; 2 UTA-RAM-One Health, Group for Universal Advance in Bioscience, Department of Food and Biotechnology Science and Engineering, Universidad Técnica de Ambato, Ambato, Ecuador; 3 Center for Genomic Medicine, Massachusetts General Hospital, Boston, MA, United States; 4 Broad Institute of MIT and Harvard, Cambridge, MA, United States; 5 Department of Pharmaceutical Sciences, Center for Health Sciences, Federal University of Paraíba, João Pessoa, Brazil; 6 Division of Infectious Diseases, Boston Children's Hospital, Harvard Medical School, Boston, MA, United States

**Keywords:** antimicrobial resistance (AMR), antimicrobial approach, chemical genomics, integrative omics, microbial genomics

## Introduction

Antimicrobial resistance (AMR) continues to erode the antibiotic arsenal that has underpinned modern medicine for eight decades (Ho et al., 2025; Patra et al., 2025; Abdallah et al., 2026). Multidrug-resistant pathogens are spreading across human medicine, veterinary practice, agriculture, and aquaculture faster than new drug classes can replace the ones they defeat (Stehling et al., 2023; Marino et al., 2025; Nazir et al., 2025; Steve et al., 2026). Meeting this challenge requires both a precise understanding of how resistance arises and evolves, and innovative strategies to circumvent or reverse it (Jacobowski et al., 2025; El-Sayed et al., 2026).

High-throughput sequencing, systems biology, analytical chemistry, and computational biology have transformed how we interrogate microbial physiology and antimicrobial response (Taj et al., 2025; Pant et al., 2026). Genomics, transcriptomics, metabolomics, proteomics, and acetyl-proteomics now reveal AMR determinants, therapeutic targets, and antimicrobial mechanisms invisible to any single technology (Luo et al., 2024; Matsumura et al., 2025; Napit et al., 2025). Paired with ecological and epidemiological methods, these tools sharpen our view of AMR as a One Health problem, in which humans, animals, and shared environments circulate resistance genes and resistant organisms alike.

This Research Topic brings together thirteen contributions (original research articles, comprehensive reviews, and a mini-review) spanning mechanistic dissection of natural compounds, genome mining for antimicrobial discovery, clinical outbreak reconstruction, environmental surveillance, and critical evaluation of alternative therapies.

## Deciphering antimicrobial mechanisms through integrative multi-omics

No single omics platform captures the full mechanistic picture of AMR. Three contributions show what emerges when these layers are combined. Wang et al. combined transcriptomics and metabolomics to show that isocorydine, a natural alkaloid, disrupts the *Mycobacterium bovis* cell envelope, triggering efflux-pump activation and lipid-metabolism reprogramming, a mechanistic lead for a pathogen that bridges veterinary and human tuberculosis. Hu et al. paired quantitative proteomics (255 differentially expressed proteins) with acetyl-proteomics (38 acetylated sites) to show that berberine sulfate acts against methicillin-resistant *Staphylococcus aureus* (MRSA) by acetylating residue K82 of the virulence regulator SarA, weakening its binding to the *agr* promoter, positioning protein acetylation as an underexplored antimicrobial target. Zhang et al. tracked *Aeromonas hydrophila* under stepwise oxytetracycline exposure mimicking aquaculture practice: resistance emerged within 3 days across three independent lineages, driven by recurrent mutations in *aheB* and *rpsJ* alongside coordinated upregulation of efflux pumps and ribosomal genes among more than 1,000 differentially expressed genes. This adaptation, the result of a coordinated genetic and transcriptional response rather than an isolated mutation, has direct implications for antibiotic dosing strategies in aquaculture, where sub-lethal drug exposure is common.

Together, these three studies convert static catalogs of resistance genes into dynamic, mechanistically grounded models of bacterial adaptation.

## Genomics and genome mining for antimicrobial discovery and epidemiology

Genome mining uncovered new biocontrol potential in two contributions. Opoku Gyamfi et al. characterized *Streptomyces melanosporofaciens* STM-2 from Taiwan, identifying 52 biosynthetic gene clusters (polyketide synthases, NRPS, RiPPs, siderophores, and terpenes) plus antifungal hydrolytic enzymes validated by AlphaFold2 structural modeling, positioning STM-2 as a promising biocontrol candidate. Reichardt et al. found that endophytic *Streptomyces* from honeybee hives carry biosynthetic potential active against both plant and honeybee pathogens, expanding the chemical space available for antimicrobial screening from an underexplored ecological niche.

A third contribution turned to genomic epidemiology in the clinic. Zhang et al. used whole-genome sequencing to trace a *Serratia marcescens* bloodstream-infection cluster across eight pediatric wards, uncovering roughly 10 months of silent intra-hospital transmission that standard surveillance had missed; bundled infection-control measures halted new cases within a month.

From mining new antibiotic gene clusters to tracing a hospital outbreak, these three studies show how far genomic tools now reach in antimicrobial research.

## Innovative therapeutic strategies beyond conventional antibiotics

One experimental study and three reviews assessed where non-traditional antimicrobial strategies stand. Wang et al. showed that hyper-branched poly-L-lysine (HBPL), a synthetic cationic polymer, inhibits MRSA (MIC 0.5 mg/mL) and biofilm formation, synergizes with levofloxacin, but is antagonistic with daptomycin, a clinically significant interaction given daptomycin's role in MRSA treatment. Resistance under HBPL pressure carried measurable fitness costs, suggesting a higher barrier than conventional antibiotics face.

Two reviews mapped where combination and peptide strategies stand. Wu et al. evaluated fosfomycin combinations against multidrug-resistant pathogens (with β-lactams, aminoglycosides, fluoroquinolones, polymyxins, and daptomycin) and traced how plasmid-borne *fos* resistance genes co-localize with extended-spectrum β-lactamase and carbapenemase genes, underscoring that combination regimens must suppress resistance co-selection, not just kill bacteria. Zhang et al. reviewed hybrid and conjugated antimicrobial peptides, showing how conjugation with antibiotics, fatty acids, photosensitizers, and nanoparticles restores activity and broadens spectrum, while flagging pharmacokinetics and protease stability as the remaining barriers to clinical translation.

A third review, by Daraghmeh et al., surveyed alternatives to antibiotics for bacterial pneumonia (phage therapy, mesenchymal stem cells, metal nanoparticles, probiotics, CRISPR-Cas, and vaccination) identifying manufacturing standardization and regulatory approval, not efficacy alone, as the largest obstacles standing between these modalities and real-world use.

Together, this experimental study and the three reviews show a rapidly diversifying antimicrobial toolbox, but one where reaching patients, not proving efficacy, remains the shared bottleneck.

## One Health perspectives: AMR surveillance, ecology, and reservoirs

Three final contributions extend this One Health lens: two examine livestock as an AMR reservoir from different angles, and a third turns to methodological reassessment for host-associated microbiome research broadly. González et al. used whole-genome sequencing and phylogenomics to characterize livestock-associated MRSA in Argentine pig farms. They identified the globally dominant CC398 lineage alongside a regional ST9 lineage not previously reported in the country. More concerning, they also detected *optrA*, a transferable gene conferring resistance to the oxazolidinone linezolid, a last-resort agent in both human and veterinary medicine. This is the first evidence of last-resort antibiotic resistance in a South American food-production environment. Where González et al. traced a single resistance gene through a single pathogen, Zhang et al. took the wider view. They applied third-generation sequencing and examined full-length 16S rRNA gene sequences from bioaerosols across an intensive dairy farm, finding *Firmicutes*- and *Proteobacteria*-dominated communities shaped primarily by UV radiation and irradiance. This reinforces the role of livestock environments as AMR-relevant microbial reservoirs more broadly.

Méndez-Sacta et al. turn the lens on methodology itself, showing how sample collection, contamination dynamics, DNA extraction, sequencing choice, and bioinformatic pipelines each bias urinary microbiome (urobiome) profiles and drive inconsistent microbiome-disease associations across studies. Their call for standardized, contamination-aware, function-oriented frameworks is a reminder (one shared with the dairy-bioaerosol findings above) that ecological inference about microbial communities is only as reliable as the methods and conditions used to generate it.

Together, these three contributions expose two distinct blind spots in AMR surveillance: resistance and reservoirs that go undetected without broad ecological coverage, and microbiome signals that shift depending on the methods used to measure them.

## Conclusions

Together, these 13 contributions show integrative microbial and chemical genomics reshaping antimicrobial science along three fronts. Multi-omics approaches resolve resistance mechanisms at a systems level that no single platform can reach alone. Genomic epidemiology reconstructs transmission chains and speeds infection-control decisions. And a rapidly diversifying therapeutic pipeline, spanning combination regimens, engineered peptides, phages, nanoparticles, and CRISPR-based tools, makes clear that no single strategy will solve AMR in isolation. Ecological and surveillance studies ground this molecular science in agricultural and environmental reality, reinforcing that durable solutions must span human medicine, animal health, and environmental stewardship.

Artificial intelligence is emerging as an accelerator across all three fronts. Integrating omics, environmental, and hydrological data into spatiotemporal predictive models could convert passive AMR surveillance into anticipatory, real-time monitoring. Such systems could detect threats in aquatic and agricultural settings before they reach the clinic (Koopmans et al., 2025; Kraemer et al., 2025; Miglietta et al., 2025; Calero-Cáceres et al., 2026). Realizing that potential will take more than algorithms: shared data infrastructure, harmonized standards, and equitable capacity-building across institutions and nations.

Antibiotics alone will not resolve AMR. The work gathered in this Research Topic advances both the mechanistic understanding and the actionable tools that a coordinated response requires.
